# Diagnostic value of three-dimensional neuroretinal rim thickness for differentiation of superior segmental optic nerve hypoplasia

**DOI:** 10.1038/s41598-023-46545-7

**Published:** 2023-11-14

**Authors:** Sooyeon Choe, Mirinae Jang, Young Kook Kim, Ki Ho Park, Jin Wook Jeoung

**Affiliations:** 1 Department of Ophthalmology, Chungnam National University College of Medicine, Daejeon, Korea; 2 Department of Ophthalmology, Chungnam National University Hospital, Daejeon, Korea; 3 Department of Ophthalmology, Yeongdong Eye Clinic, Pohang, Korea; 4 Department of Ophthalmology, Seoul National University Hospital, Seoul National University College of Medicine, 101 Daehak-ro, Jongno-gu, Seoul, 03080 Korea

**Keywords:** Eye diseases, Medical research

## Abstract

Little is known about the diagnostic utility of three-dimensional neuroretinal rim thickness (3D-NRRT) for differentiating patients with superior segmental optic nerve hypoplasia (SSOH) from normal-tension glaucoma (NTG). Since SSOH is defined by characteristic optic nerve head features, investigation of diagnostic usefulness of 3D-NRRT is necessary. In this cross-sectional study, 49 SSOH eyes, 52 NTG eyes, and 41 normal eyes were enrolled. Retinal nerve fiber layer thickness (RNFLT) and 3D-NRRT values, as obtained in the right-eye orientation by optical coherence tomography (OCT), were recorded. On RNFLT clock-hour comparison, the 11–3 clock-hour sectors were significantly thinner for SSOH than for NTG (all *P* < 0.01). As for 3D-NRRT, whereas the 1 and 2 sectors were significantly thinner for SSOH (*P* < 0.001, *P* = 0.004), the 6–11 sectors were significantly thinner for NTG (all *P* < 0.01). The area under receiver operating characteristic (AUROC) curves of the superior and nasal quadrants of RNFLT (0.838, 0.729) were significantly greater than those of 3D-NRRT (0.518, 0.588; *P* < 0.001, *P* = 0.043). However, the AUROCs of the inferior and temporal quadrants were significantly greater for 3D-NRRT (0.728, 0.760) than for RNFLT (0.527, 0.550; *P* = 0.008, *P* = 0.019). The appropriate use of 3D-NRRT can be useful in differentiating SSOH from NTG.

## Introduction

Superior segmental optic hypoplasia (SSOH) is a developmental anomaly manifesting as relative hypoplasia of the superior part of the optic nerve head and retinal nerve fiber layer (RNFL). It is characterized by four findings: (1) pallor of the superior optic disc, (2) thinning of the superior RNFL correspondent with optic disc pallor, (3) relatively superior entrance of central retinal vessels, and (4) superior peripapillary scleral halo^1–3^.

Notwithstanding these characteristic findings, SSOH often is difficult to differentiate from glaucoma, especially normal-tension glaucoma (NTG), because NTG also shows focal thinning of the neuroretinal rim (NRR) of the optic disc as well as sector radial thinning of the RNFL^4^. Moreover, only about half of cases or less show definite features of SSOH^5^. In terms of clinical course, NTG usually follows a progressive and chronic condition, while SSOH is generally considered a non-progressive, congenital anomaly^1,6^. Therefore, differentiation between SSOH and NTG, especially with superior RNFL defect, is important but challenging issue^6^.

Some efforts have been exerted previously to diagnose SSOH from NTG. Yamada et al.^7^ and Han et al.^8^ analyzed RNFL thickness (RNFLT) using optical coherence tomography (OCT) to differentiate these two diseases. Lee et al.^9^ compared peripapillary vessel density between SSOH and NTG using OCT angiography. When SSOH is defined by its characteristic features of optic nerve head and excellent diagnostic performance of NRR parameters for glaucoma have been previously proved, to the best of our knowledge, there are few studies comparing SSOH and NTG in terms of NRR thickness. Thus, the purpose of the present study was to apply three-dimensional neuroretinal rim thickness (3D-NRRT) to the differential diagnosis of SSOH and NTG patients using OCT and to compare its utility in this regard with that of RNFLT.

## Results

### Demographic and clinical characteristics of study subjects

A total of 49 SSOH eyes (49 subjects), 52 severity-matched NTG eyes (52 subjects) and 41 normal eyes of 41 age-matched patients meeting the eligibility criteria were included in this study. Table 1 summarizes the demographic and ocular characteristics of each group. There were no significant inter-group differences in ocular characteristics by sex, IOP or refractive error (*P* = 0.051, *P* = 0.179, *P* = 0.081). Age at diagnosis showed no significant difference between the SSOH group (36.02 ± 15.5 years) and the control group (36.05 ± 9.3 years); however, the average age at diagnosis of the NTG group (48.06 ± 12.7 years) was significantly older than those of the SSOH and control groups (*P* < 0.001). There was no significant difference in mean deviation of VF between the SSOH (− 2.77 ± 3.25 dB) and NTG (− 2.03 ± 2.05 dB) groups.

**Table 1:** Clinical and demographic characteristics.

	Normal (n = 41)	SSOH (n = 49)	NTG (n = 52)	*P* value	Post hoc analysis		
					A–B	A–C	B–C
Age at diagnosis, year	36.05 ± 9.3	36.02 ± 15.5	48.06 ± 12.7	< 0.001*	0.988	** < 0.001**	** < 0.001**
Female, N (%)	22 (53.7)	36 (73.5)	32 (61.5)	0.051^†^			
IOP, mmHg	14.02 ± 2.92	13.91 ± 2.40	13.12 ± 2.58	0.179*			
SE, D	− 3.63 ± 3.02	− 3.83 ± 3.28	− 2.31 ± 3.18	0.081*			
MD of VF, dB	− 0.03 ± 1.41	− 2.77 ± 3.25	− 2.03 ± 2.05	< 0.001*	** < 0.001**	** < 0.004**	0.333

Mean ± standard deviation. Statistically significant values are shown in bold.

*IOP* intraocular pressure, *SE* spherical equivalent, *MD of VF* mean deviation of visual field, *A* normal, *B* SSOH, *C* NTG.

*Comparison was performed using one-way ANOVA with post hoc Scheffe’s multiple comparison testing.

^†^Comparison was performed using χ^2^ test.

### Comparison of RNFLT and 3D-NRRT among SSOH, NTG and normal subjects

Tables 2 and 3 show the mean values of the average, 4-quadrant and 12-clock-hour-sectoral RNFLT and 3D-NRRT measurements (along with comparative statistics) among the groups.

**Table 2:** Comparison of RNFLT among clock hours between groups.

OCT Parameters (μm)	Normal (n = 41)	SSOH (n = 49)	NTG (n = 52)	*P* value*	Post hoc analysis		
					A–B	A–C	B–C
Average	93.7 ± 8.3	74.6 ± 9.9	80.1 ± 9.5	** < 0.001**	** < 0.001**	** < 0.001**	**0.015**
Quadrant							
Superior	114.7 ± 15.3	67.8 ± 12.8	84.3 ± 13.3	** < 0.001**	** < 0.001**	** < 0.001**	** < 0.001**
Nasal	67.68 ± 10.1	53.4 ± 10.3	62.1 ± 9.6	** < 0.001**	** < 0.001**	**0.030**	** < 0.001**
Inferior	118.4 ± 14.8	107.8 ± 18.7	106.9 ± 17.1	**0.003**	**0.015**	**0.006**	0.960
Temporal	74.9 ± 11.7	66.9 ± 15.3	66.9 ± 12.3	**0.006**	**0.018**	**0.016**	1.000
Clock hours							
10	87.4 ± 16.0	73.5 ± 20.1	70.6 ± 17.1	** < 0.001**	**0.002**	** < 0.001**	0.710
11	114.7 ± 15.3	67.8 ± 12.8	76.5 ± 20.3	** < 0.001**	** < 0.001**	** < 0.001**	**0.005**
12	111.2 ± 28.8	59.1 ± 14.2	86.9 ± 21.6	** < 0.001**	** < 0.001**	** < 0.001**	** < 0.001**
1	100.7 ± 24.0	56.0 ± 14.4	89.3 ± 20.5	** < 0.001**	** < 0.001**	**0.024**	** < 0.001**
2	81.0 ± 18.3	55.3 ± 13.1	70.5 ± 13.8	** < 0.001**	** < 0.001**	**0.004**	** < 0.001**
3	59.1 ± 12.6	49.7 ± 10.1	56.4 ± 9.2	** < 0.001**	** < 0.001**	0.477	**0.007**
4	62.0 ± 9.2	54.8 ± 10.1	59.3 ± 11.7	**0.005**	**0.006**	0.468	0.101
5	89.0 ± 17.5	78.8 ± 20.8	83.4 ± 18.3	**0.042**	**0.042**	0.370	0.478
6	119.8 ± 21.7	109.9 ± 28.0	110.9 ± 25.0	0.136	0.185	0.245	0.981
7	144.9 ± 20.5	134.6 ± 22.4	126.4 ± 25.1	**0.001**	0.106	**0.001**	0.201
8	80.6 ± 15.8	78.9 ± 22.2	75.5 ± 18.4	0.423	0.915	0.447	0.675
9	56.5 ± 9.8	54.2 ± 11.0	55.7 ± 10.9	0.582	0.595	0.929	0.793

Mean ± standard deviation. Statistically significant values are shown in bold.

*A* normal, *B* SSOH, *C* NTG.

*Comparison was performed using one-way ANOVA with post hoc Scheffe’s multiple comparison testing.

**Table 3:** Comparison of 3D-NRRT among clock hours between groups.

OCT Parameters (μm)	Normal (n = 41)	SSOH (n = 49)	NTG (n = 52)	*P* value*	Post hoc analysis		
					A–B	A–C	B–C
Average	485.2 ± 53.7	256.8 ± 47.9	230.9 ± 55.9	** < 0.001**	** < 0.001**	**0.042**	0.051
Quadrant							
Superior	293.9 ± 55.4	209.7 ± 48.8	203.8 ± 53.4	** < 0.001**	** < 0.001**	** < 0.001**	0.855
Nasal	294.2 ± 91.4	237.5 ± 71.1	262.1 ± 85.7	**0.006**	**0.006**	0.183	0.329
Inferior	331.2 ± 63.1	350.5 ± 70.8	283.4 ± 75.1	** < 0.001**	0.433	** < 0.001**	0.433
Temporal	221.5 ± 49.1	229.7 ± 53.8	174.1 ± 56.1	** < 0.001**	0.769	** < 0.001**	** < 0.001**
Clock hours							
10	226.4 ± 47.5	228.9 ± 60.5	171.7 ± 61.3	** < 0.001**	0.980	** < 0.001**	** < 0.001**
11	276.1 ± 46.3	236.2 ± 60.4	169.6 ± 62.7	** < 0.001**	**0.006**	** < 0.001**	** < 0.001**
12	307.8 ± 65.6	212.9 ± 54.7	206.4 ± 64.1	** < 0.001**	** < 0.001**	** < 0.001**	0.868
1	297.7 ± 77.4	180.0 ± 58.2	235.6 ± 62.1	** < 0.001**	** < 0.001**	** < 0.001**	** < 0.001**
2	290.4 ± 100.2	183.9 ± 72.7	239.0 ± 75.9	** < 0.001**	** < 0.001**	**0.014**	**0.004**
3	279.1 ± 98.8	228.1 ± 80.3	244.6 ± 91.4	**0.028**	**0.030**	0.191	0.654
4	313.1 ± 87.9	300.4 ± 77.5	302.6 ± 103.5	0.784	0.804	0.859	0.992
5	345.9 ± 75.6	359.3 ± 83.3	326.3 ± 99.6	0.167	0.772	0.564	0.171
6	351.5 ± 71.1	382.9 ± 79.5	301.2 ± 0.7	** < 0.001**	0.164	**0.010**	** < 0.001**
7	296.1 ± 62.5	309.2 ± 85.8	222.8 ± 78.5	** < 0.001**	0.722	** < 0.001**	** < 0.001**
8	229.4 ± 55.0	237.7 ± 58.8	181.4 ± 60.1	** < 0.001**	0.794	**0.001**	** < 0.001**
9	208.7 ± 55.4	222.5 ± 59.1	169.3 ± 57.7	** < 0.001**	0.531	**0.006**	** < 0.001**

Mean ± standard deviation. Statistically significant values are shown in bold.

*A* normal, *B* SSOH, *C* NTG.

*Comparison was performed using one-way ANOVA with post hoc Scheffe’s multiple comparison testing.

**Figure 1:**
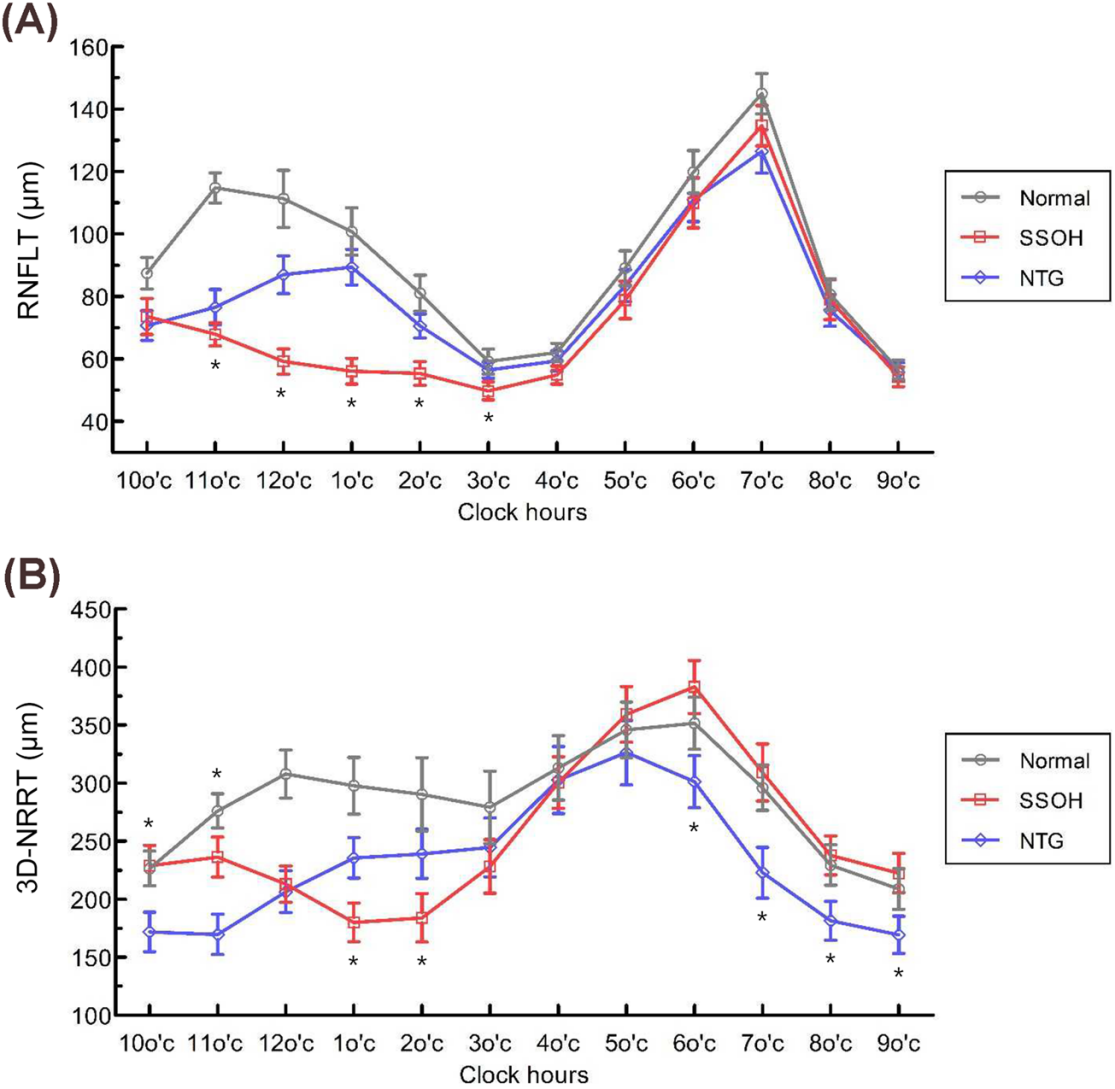
Clock-hour distribution of RNFLT and 3D-NRRT between groups. *Statistically significant difference between group B (SSOH) and group C (NTG).

The average RNFLT was greatest, significantly, in the normal group (93.7 ± 8.3 μm, *P* < 0.001), followed by the NTG (80.1 ± 9.5 μm) and SSOH (74.6 ± 9.9 μm) groups. There was a significant difference in average RNFLT between the SSOH and NTG groups (*P* = 0.015). In the quadrant analysis, the superior and nasal RNFLTs of the NTG group were significantly greater than that of the SSOH group (both *P* < 0.001). In the clock-hour analysis, the RNFLTs from the 11–3 o’clock sectors were revealed to be thicker in the NTG group than in the SSOH group (Table 2 and Fig. 1A).

As for the 3D-NRRT, the average was significantly greater in the normal group (485.2 ± 53.7 μm, *P* < 0.001) than in the SSOH (256.8 ± 47.9 μm) or NTG (230.9 ± 55.9 μm) group. However, there was marginal difference in average 3D-NRRT between the SSOH and NTG (*P* = 0.051) groups. In contrast to the RNFLT case, the average temporal 3D-NRRT of the SSOH group was greater than that of the NTG group (*P* < 0.001), and there were no significant differences between those two groups in the superior, nasal or inferior quadrant. In the clock-hour analysis, the 3D-NRRTs from the 6–11 o’clock sectors were greater in the SSOH group (all *P* < 0.001), whereas those from the 1 and 2 o’clock sectors were greater in the NTG group (*P* < 0.001, *P* = 0.004) (Table 3 and Fig. 1B).

### ROC analysis

Table 4 provides the data on the area under receiver operating characteristic (AUROC) curve comparison between RNFLT and 3D-NRRT for differential diagnosis of SSOH from NTG. There was no significant difference between average RNFLT (0.668) and average 3D-NRRT (0.628, *P* = 0.630). The AUROCs of the superior and nasal quadrants of RNFLT (0.838, 0.729) were significantly greater than those of 3D-NRRT (0.518, 0.588, *P* < 0.001, *P* = 0.043). However, the inferior and temporal quadrants were significantly greater for 3D-NRRT (0.728, 0.760) than for RNFLT (0.527, 0.550, *P* = 0.008, *P* = 0.019). In the clock-hour analysis, whereas the AUROC value of 3D-NRRT was greater than that of RNFLT in the 6 to 10 o’clock sectors (all *P* < 0.01), the AUROC value of RNFLT was greater than that of 3D-NRRT in the 12, 1 and 3 o’clock sectors (all *P* < 0.05).

**Table 4:** AUROC curve comparison between RNFLT and 3D-NRRT for differential diagnosis of SSOH from NTG.

	RNFLT		3D-NRRT		*P* value
	AUROC (95% CI)	Best cut-off* (sensitivity, specificity)	AUROC (95% CI)	Best cut-off* (sensitivity, specificity)	
Average	0.668 (0.567–0.758)	≤ 85.5 (91.8, 36.5)	0.628 (0.526–0.722)	> 218.5 (79.6, 48.1)	0.630
Quadrant					
Superior	**0.838 (0.751–0.904)**	** ≤ 74.5 (73.5, 82.7)**	0.518 (0.417–0.619)	> 250.4 (8.2, 76.9)	** < 0.001**
Nasal	**0.729 (0.632–0.813)**	** ≤ 53.5 (59.2, 78.9)**	0.588 (0.486–0.685)	≤ 233.2 (55.1, 63.5)	**0.043**
Inferior	0.527 (0.426–0.628)	> 111.5 (44.9, 65.4)	**0.728 (0.631–0.812)**	** > 269.8 (91.8, 48.1)**	**0.008**
Temporal	0.550 (0.448–0.649)	≤ 54.5 (22.5, 90.4)	**0.760 (0.665–0.840)**	** > 183.7 (81.6, 63.5)**	**0.019**
Clock hours					
10	0.531 (0.429–0.631)	> 49.5 (93.9, 17.3)	**0.760 (0.665–0.839)**	** > 207.6 (67.4, 76.9)**	** < 0.001**
11	0.669 (0.569–0.760)	> 99.5 (40.8, 88.5)	0.783 (0.690–0.859)	> 198.7 (75.5, 73.1)	0.054
12	**0.847 (0.761–0.911)**	** ≤ 70.5 (75.0, 76.9)**	0.549 (0.447–0.649)	> 164.0 (83.7, 34.6)	** < 0.001**
1	**0.911 (0.838–0.959)**	** ≤ 64.5 (81.6, 88.5)**	0.740 (0.643–0.822)	≤ 190.5 (61.2, 78.9)	** < 0.001**
2	0.790 (0.698–0.865)	≤ 59.5 (67.4, 76.9)	0.712 (0.613–0.797)	≤ 190.4 (59.2, 79.9)	0.183
3	**0.710 (0.611–0.796)**	** ≤ 48.5 (61.2, 82.7)**	0.551 (0.449–0.650)	≤ 243.3 (65.3, 51.9)	**0.041**
4	0.602 (0.500–0.698)	≤ 53.5 (53.1, 69.2)	0.524 (0.422–0.624)	> 292.3 (53.1, 57.7)	0.297
5	0.552 (0.450–0.651)	≤ 64.5 (34.7, 88.5)	0.630 (0.528–0.724)	> 271.0 (89.8, 36.5)	0.319
6	0.506 (0.405–0.607)	> 134.5 (24.5, 88.5)	**0.766 (0.671–0.844)**	** > 361.7 (57.1, 84.6)**	** < 0.001**
7	0.590 (0.488–0.687)	> 102.5 (95.9, 25.0)	**0.782 (0.689–0.858)**	** > 228.4 (85.7, 59.6)**	**0.002**
8	0.536 (0.434–0.635)	> 71.5 (57.1, 55.8)	**0.762 (0.667–0.841)**	** > 220.1 (67.4, 80.8)**	** < 0.001**
9	0.542 (0.440–0.642)	≤ 57.5 (65.3, 46.2)	**0.753 (0.547–0.833)**	** > 195.9 (71.4, 73.1)**	**0.001**

Statistically significant values are shown in bold.

*CI* confidence interval.

*The best cut-off value was selected using the Youden index value that maximized the value of ‘sensitivity + specificity − 1’.

**Figure 2:**
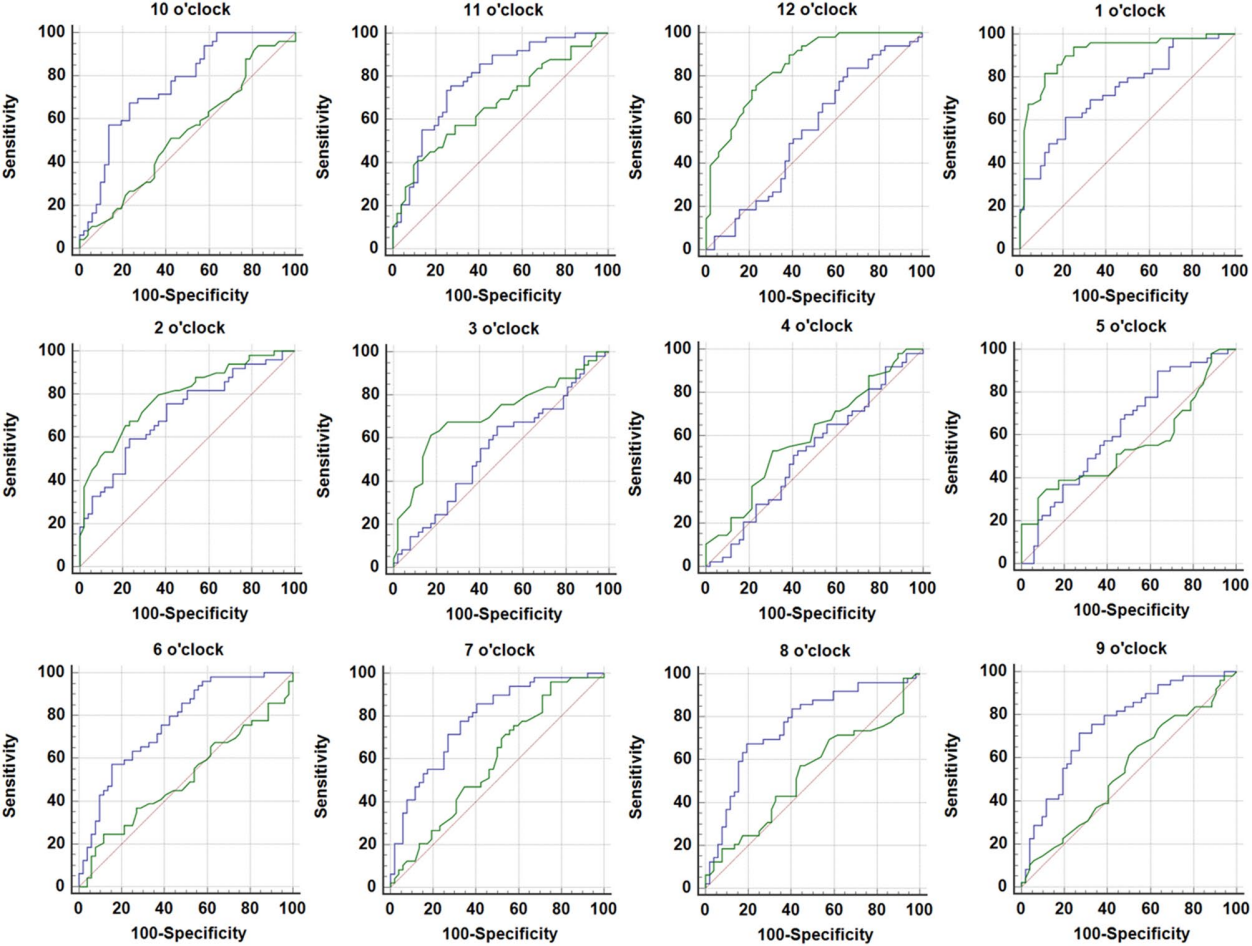
ROC curves of 3D-NRRT (blue-line) and RNFLT (green-line).

The sensitivity and specificity values as determined by the best cut-off values ranged from 24.5 to 93.9% and from 17.3 to 88.5%, respectively, for RNFLT, and from 53.1 to 89.8% and 34.6 to 84.6%, respectively, for 3D-NRRT (Table 4). Figure 2 plots the AUROC curves of RNFLT and 3D-NRRT by clock-hour sector for discrimination of SSOH from NTG.

### Sensitivity and specificity for discrimination of SSOH from NTG

In the OCT RNFL measurements, the superior quadrant showed high sensitivity (73.4% at 80% specificity, 55.1% at 90% specificity) and high specificity (73.1% at 80% sensitivity, 50.0% at 90% sensitivity). In the 3D-NRRTs, the temporal quadrant showed high sensitivity (53.1% at 80% specificity, 36.7% at 90% specificity) and high specificity (63.4% at 80% sensitivity, 36.5% at 90% sensitivity). In the clock-hour analysis, high sensitivity (85.7% at 80% specificity, 71.4% at 90% specificity) and specificity (88.5% at 80% sensitivity, 75.0% at 90% sensitivity) were obtained for RNFLT in the 1 o’clock sector. By contrast, high sensitivity and specificity 3D-NRRT values were observed in the 6 to 11 o’clock sectors (Table 5).

**Table 5:** Sensitivity and specificity for discrimination of SSOH from NTG.

		RNFLT				3D-NRRT			
		Sensitivity (%)		Specificity (%)		Sensitivity (%)		Specificity (%)	
		80% SP	90% SP	80% SE	90% SE	80% SP	90% SP	80% SE	90% SE
Average		34.7	22.5	40.4	36.5	26.5	16.3	48.1	36.5
Quadrant									
Superior	73.4		55.1	73.1	50.0	10.2	8.2	25.0	17.3
Nasal	55.1		36.7	50.0	26.9	30.6	20.4	32.7	19.2
Inferior	22.5		12.2	23.1	1.9	46.9	24.5	53.6	18.1
Temporal	26.5		22.5	30.8	5.8	53.1	36.7	63.4	36.5
Clock hours									
10	18.4		10.2	23.1	19.2	59.2	30.6	55.8	42.3
11	44.9		38.8	36.5	17.3	57.1	30.6	65.4	42.3
12	69.2		59.6	65.3	49.0	18.4	6.1	34.6	19.2
1	85.7		71.4	88.5	75.0	51.0	38.8	42.3	30.8
2	61.2		51.0	63.5	36.5	42.9	34.7	50.0	28.8
3	63.3		36.7	38.5	15.4	24.5	14.3	19.4	11.5
4	26.5		14.3	25.0	15.4	20.4	4.1	25.0	17.3
5	38.8		34.7	17.3	13.5	36.7	22.5	42.3	36.5
6	24.5		20.4	11.5	1.9	57.1	42.9	57.7	46.2
7	26.5		12.2	32.7	28.8	55.1	40.8	64.5	44.2
8	24.5		18.4	11.5	7.6	67.4	36.7	59.6	40.4
9	22.4		14.3	28.8	9.6	55.1	32.7	55.8	36.5

*SE* sensitivity, *SP* specificity.

## Discussion

This study demonstrated that both and 3D-NRRT and RNFLT can be useful in differential diagnosis of SSOH and NTG. In the case of 3D-NRRT, the values for SSOH from 6 to 11 o’clock were significantly greater than those for NTG, but in the 1 and 2 o’clock sectors, the SSOH values were significantly thinner than the NTG ones. This means that it may be helpful to differentiate SSOH from NTG by 3D-NRRT, additionally to RNFLT, the well-known diagnostic parameter. Yamamoto et al.^11^ found NRR thinning on the superior nasal side and corresponding RNFL thinning in SSOH to be the most prominent, which is consistent with the results of the present study.

In comparing RNFLT, SSOH thickness was significantly thinner than that in NTG from 11 to 3 o’clock. The AUROC values also showed that RNFLT from 11 to 3 o’clock was generally higher in discriminating power for the two diseases. In particular, AUROC values for RNFLT at 12 and 1 o'clock are expected to be clinically useful in differentiating SSOH from NTG. Similar conclusions have been reached in several studies using RNFLT to determine the differences between NTG and SSOH or SSOH and normal eyes. For example, Yamada et al.^7^ reported that RNFLT in SSOH was significantly thinner than that in NTG in the 12–2 o’clock sectors. Han et al.^8^ reported that RNFLT in SSOH was thinner than that in NTG in the 1–2 o’clock sectors. When we set the models that combined the 7 o’clock 3D-NRRT (which showed the greatest AUROC among the NRRT values), the AUROC improved to 0.942 (1 o’clock RNFLT + 7 o’ clock 3D-NRRT) and the AUROC improved to 0.954 (1 o’clock RNFLT + 12 o’clock RNFLT + 7 o’ clock 3D-NRRT). Certainly, using inferotemporal 3D-NRRT can provide supplemental information S1 for differentiation of SSOH and NTG. This suggestion should be further verified with a larger number of samples in the future.

When we discriminated SSOH and NTG from the normal controls in the present study, the results corresponded well with those of earlier studies. As indicated in Supplementary Table S1, SSOH was best differentiated when using the 12–1 o’clock sectors (superior segment), whereas, as shown in Supplementary Table S2, NTG was best diagnosed with the 11 o’clock sector (superotemporal segment). The addition of inferior sectors did not further increase the AUROC values when discriminating SSOH from the normal controls.

**Figure 3:**
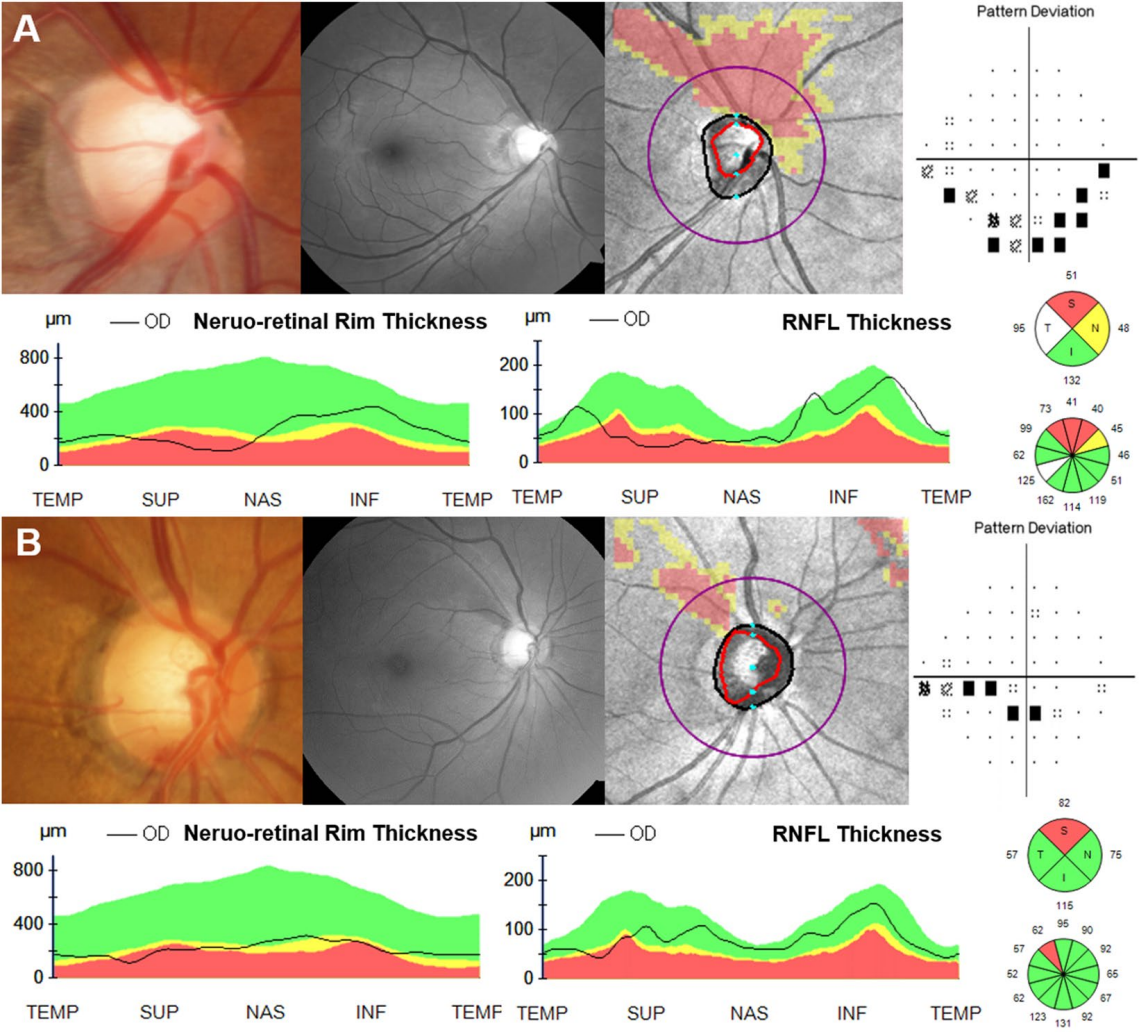
Representative cases of SSOH and NTG. (**A**) Right eye of 35-year-old female diagnosed with SSOH (Best-corrected visual acuity [BCVA] (logMAR) 0, IOP 13 mmHg, axial length 24.99 mm, SE − 5.125D). Disc stereo-photography and red-free fundus photography showed superonasal disc rim narrowing and superior RNFL defect. HVF showed inferior arcuate visual field (VF) loss (MD − 4.67 dB). Optic disc cube scan and RNFLT map showed superior RNFL thinning. 3D-NRRT map showed superior to nasal 3D-NRR thinning. (**B**) Right eye of 51-year-old female diagnosed with NTG (BCVA (logMAR) 0, IOP 12 mmHg, axial length 25.03 mmHg, SE + 0.625D). Disc stereo-photography and red-free fundus photography showed superotemporal disc rim narrowing and superotemporal RNFL defect. HVF showed inferior arcuate VF loss (MD − 3.2 dB). Optic disc cube scan and RNFLT map showed superotemporal RNFL thinning. The 3D-NRRT map showed superior and inferior 3D-NRR borderline thinning.

The two cases shown in Fig. 3 demonstrate the characteristics of the RNFLT and 3D-NRRT described above. The patient with SSOH has a thin superior RNFL, a thin superonasal NRR and a normal inferior NRR; however, the patient with NTG has a thin superotemporal NRR and RNFL as well as a thin inferior NRR. Ratanawongphaibul et al.^13^ derived the minimum distance band (MDB) thickness, which is a 3D-NRR parameter calculated by custom-built software from a high-density optic nerve volume scan, and reported that this global MDB thickness detected glaucoma progression earlier than either than global RNFLT. This means that 3D-NRRT change may precede RNFLT change. On this basis, further longitudinal studies on SSOH are called for to further elucidate the role of NRR and RNFLT.

Spectral-domain optical coherence tomography (SD-OCT) measures quantitative and structural intraocular parameters^14^, and so it has been deemed essential to use the two-dimensional (2D) peripapillary RNFLT utility provided by SD-OCT when diagnosing or differentially diagnosing glaucoma^15^. However, several recent studies have demonstrated that 3D NRR volume scans are less likely to have clinically significant artifacts than are 2D peripapillary RNFLT scans, for both glaucomatous and normal eyes^16,17^. Tai et al.^18^ compared the clock-hour-sectoral thicknesses of NRR and RNFL to differentiate between preperimetric glaucoma and normal eyes. They reported that larger AUROCs for 3D-NRRT were observed at 6 and 7 o’clock than for RNFLT. Kim et al.^10^, reporting on the diagnostic accuracy of 3D-NRRT for differentiation of myopic glaucoma from myopia, demonstrated that 3D-NRRT measurement reduced the false-positive rate for glaucoma diagnosis and enhanced the accuracy of glaucoma detection in myopic eyes. In line with these findings, our results suggest that measurement of 3D-NRRT, especially in the inferotemporal segment, can be complementary to that of RNFLT for differentiation of SSOH from NTG. However, the superior and nasal segments of 3D-NRRT showed relatively low AUROC compared to RNFLT (*P*’s < 0.05). The potential influence of the superior peripapillary scleral halo in SSOH eyes on 3D-NRRT measurements should be considered. Hence, caution is advised when utilizing superonasal 3D-NRRT for diagnosing SSOH.

The present study has some potential limitations. First, the NTG subjects were significantly older than their SSOH counterparts. However, we mitigated the potential bias resulting from the impact of age-related changes in RNFLT^18^ by selecting subjects in a VF-severity-matched manner^19^. Second, although there was no significant difference, the SSOH group had a more myopic value of SE. Further research with well-matched controls is warranted. Third, various optic disc morphologies and forms of parapapillary atrophy may affect the NRR and RNFL structures of NTG patients, and as such, more detailed complementary studies are needed to classify and structurally analyze NTG patients according to the optic disc and parapapillary structures. Lastly, the enrolled NTG participants were predominantly younger (mean age 48.1 years old) and had early-stage glaucoma (mean MD − 2.03 dB). Also, we included cases presenting superior optic disc damage, a condition that poses a challenge in distinguishing it from SSOH. Thus, caution is warranted in generalizing the results of this study to all NTG patients.

In conclusion, both RNFLT and 3D-NRRT may be helpful in differentiating SSOH patients from NTG. Further prospective longitudinal studies on SSOH patients’ clinical course and structural changes are warranted. It will be interesting to see if they corroborate and add relevance to the present data.

## Methods

This cross-sectional study was approved by the Seoul National University Hospital Institutional Review Board (IRB No. 2107-134-1236) and faithfully adhered to the tenets of the Declaration of Helsinki. Written informed consent was waived due to the retrospective nature of the study by the Institutional Review Board of Seoul National University Hospital.

### Study subjects

Normal subjects as well as SSOH and NTG patients were enrolled from Seoul National University Hospital Glaucoma Clinic in Seoul, Korea. All subjects underwent a comprehensive ophthalmic examination, including visual acuity (VA) assessment, slit-lamp biomicroscopy, gonioscopy, Goldmann applanation tonometry (Haag-Streit, Koniz, Switzerland), refraction, dilated fundus examination, disc stereo-photography, red-free fundus photography by digital fundus camera (VISUCAM, Carl Zeiss Meditec, Inc., Jena, Germany), Cirrus HD-OCT (software version 6.0; Carl Zeiss Meditec, Inc.), and standard automated perimetry (Humphrey C 24-2 SITA Standard visual field [HVF]; Carl Zeiss Meditec, Inc.). Based on a retrospective review of medical records, eligible participants were consecutively enrolled.

SSOH was defined according to the following criteria: (1) the presence of at least 2 of the characteristic features of SSOH, including a relatively superior entrance of the central retinal artery, thinning of the superior RNFL, a superior peripapillary scleral halo, and superior optic disc pallor; (2) visual field (VF) test findings of inferior altitudinal or sector-like defects; (3) no VF progression during at least 2 years of follow-up; (4) peak intraocular pressure (IOP) ≤ 21 mmHg on Goldmann applanation tonometry; (5) no history of refractive surgeries; (6) absence of coexisting NTG, and (7) absence of other diseases affecting the VA or VF. For patients with bilateral SSOH, one eye was randomly selected.

We defined NTG with superior RNFL thinning based on the following criteria: (1) glaucomatous optic nerve damage (narrowing or notching of the superior or superotemporal NRR); (2) superotemporal RNFL defects corresponding to glaucomatous changes of the optic nerve head but with relatively intact inferotemporal RNFL; (3) glaucomatous VF defects corresponding to glaucomatous changes of the optic nerve head or RNFL defects; (4) open angle on gonioscopy; (5) baseline IOP ≤ 21 mmHg with Goldmann applanation tonometry; (6) no history of refractive surgeries, and (7) absence of other diseases affecting the VA or VF. For patients with bilateral NTG with inferior VF defects, one eye was randomly selected. Also, we excluded cases that raised suspicions of presenting characteristics associated with both SSOH and NTG. We matched the baseline severity of NTG and SSOH cases based on the mean deviation of HVF test results.

The inclusion criteria for the control group were: (1) IOP < 21 mmHg without antiglaucoma medication; (2) normal-appearing optic discs without any RNFL defect, and (3) normal VFs. A normal-appearing optic disc was defined as the absence of glaucomatous optic neuropathy and pallor or swelling of the optic disc. A normal VF was defined as the absence of glaucomatous VF defects and neurological-field defects. The healthy control patients thus selected were matched with the SSOH group in terms of age and refraction (spherical equivalence [SE]).

### Measurement of RNFLT and 3D-NRRT

After obtaining 200X200 optic disc cube scans, the RNFLTs were automatically measured by Cirrus HD-OCT with the built-in analysis algorithm (software version 6.0; Carl Zeiss Meditec, Inc.), according to the following parameters: peripapillary average, four quadrants (superior, nasal, inferior, and temporal); 12 clock-hour-sectoral RNFLTs.

Measurements of 3D-NRRT^10^, defined as the distance between the Bruch’s membrane opening and vitreoretinal interface (which is associated with the minimum cross-sectional rim area in the given direction), were extracted from the device and converted to average, four quadrant (superior, nasal, inferior and temporal), and 12 clock-hour-sectoral values. The right-eye orientation was used in documenting and analyzing the OCT data.

### Statistical analysis

Continuous variables were compared among the three groups by one-way ANOVA with Scheffe’s post hoc analysis. Categorical data were compared using the χ^2^ test. The diagnostic performances of the RNFLTs and 3D-NRRTs for detection of myopic glaucoma were determined by calculating the AUROC curves. The best cut-off value was selected according to the Youden index value (which maximizes the value of ‘sensitivity + specificity − 1’)^1^ In all of the analyses, parametric or nonparametric tests were used based on the normality test, and the 95% confidence interval (CI) was calculated. A statistical analysis was performed using the SPSS statistical package (SPSS for Windows, version 19.0, IBM-SPSS, Chicago, IL, USA), and a P value less than 0.05 was considered statistically significant. A receiver operating characteristic (ROC) analysis was performed using Medcalc (version 20.009; MedCalc software Ltd., Ostend, Belgium).

### Supplementary Information

Supplementary Tables.

## Data Availability

The datasets analysed during the current study are not publicly available due to patient data privacy policy, but are available from the corresponding author (J.W.J.) on reasonable request.

## Abbreviations

AUROC: Area under receiver operating characteristic
NRR: Neuroretinal rim
NTG: Normal-tension glaucoma
OCT: Optical coherence tomography
RNFL: Retinal nerve fiber layer
RNFLT: Retinal nerve fiber layer thickness
ROC: Receiver operating characteristic
SE: Spherical equivalence
SSOH: Superior segmental optic hypoplasia
3D-NRRT: Three-dimensional neuroretinal rim thickness
VA: Visual acuity
VF: Visual field
