# Supplementary material for: Diagnostic value of three-dimensional neuroretinal rim thickness for differentiation of superior segmental optic nerve hypoplasia
Source: Sci Rep. 2023 Nov 14;13:19877. doi: 10.1038/s41598-023-46545-7 (PMC10645813; doi:10.1038/s41598-023-46545-7)
Supplement: Supplementary file 1 — Supplementary Tables. [file 41598_2023_46545_MOESM1_ESM.pdf]

Supplementary Information

Diagnostic Value of Three-dimensional Neuroretinal Rim Thickness for Differentiation of Superior Segmental Optic Nerve Hypoplasia

Sooyeon Choe, MD[1,2], Mirinae Jang, MD[3], Young Kook Kim, MD, PhD[4,5], Ki Ho Park, MD, PhD[4,5] and Jin Wook Jeoung, MD, PhD[4,5]

[1] Department of Ophthalmology, Chungnam National University College of Medicine, Daejeon, Korea

[2] Department of Ophthalmology, Chungnam National University Hospital, Daejeon, Korea

[3] Department of Ophthalmology, Yeongdong Eye Clinic, Pohang, Korea

[4] Department of Ophthalmology, Seoul National University College of Medicine, Seoul, Korea

[5] Department of Ophthalmology, Seoul National University Hospital, Seoul, Korea

Supplementary Table S1. AUROC Curve Comparison of RNFLT and 3D-NRRT for Differential Diagnosis of SSOH from Normal Eyes

Supplementary Table S2. AUROC Curve Comparison of RNFLT and 3D-NRRT for Differential Diagnosis of NTG from Normal Eyes

Supplementary Table S1. AUROC Curve Comparison of RNFLT and 3D-NRRT for Differential Diagnosis of SSOH from Normal Eyes

	**RNFLT**		**3D-NRRT**	
	**AUROC (95% CI)**	**Best Cut-Off[a] (Sensitivity, Specificity)**	**AUROC (95% CI)**	**Best Cut-Off[a] (Sensitivity, Specificity)**
**Average**	0.947 (0.878-0.983)	≤85 (89.8, 90.2)	0.642 (0.534-0.740)	≤242.8 (40.8, 82.9)
**Quadrant**				
Superior	0.995 (0.950-1.000)	≤87 (93.9, 97.6)	0.884 (0.799-0.942)	≤250.4 (89.8, 73.2)
Nasal	0.843 (0.751-0.911)	≤53 (59.2, 100.0)	0.673 (0.566-0.768)	≤264.7 (69.4, 61.0)
Inferior	0.651 (0.544-0.749)	≤105 (44.9, 85.4)	0.575 (0.466-0.679)	>315.4 (69.4, 46.3)
Temporal	0.693 (0.587-0.786)	≤69 (63.3, 68.3)	0.564 (0.456-0.669)	>231.8 (51.0, 70.7)
**Clock hours**				
10	0.724 (0.620-0.813)	≤79 (67.4, 68.3)	0.504 (0.397-0.611)	≤164.9 (20.4, 92.7)
11	0.912 (0.834-0.962)	≤116 (85.7, 82.9)	0.710 (0.605-0.801)	≤221.8 (42.9, 90.2)
12	0.963 (0.901-0.992)	≤83 (98.0, 82.9)	0.879 (0.793-0.938)	≤244.3 (77.6, 87.8)
1	0.957 (0.892-0.988)	≤70 (89.8, 92.7)	0.905 (0.825-0.957)	≤204.2 (69.4, 95.1)
2	0893 (0.810-0.948)	≤57 (65.3, 100.0)	0.810 (0.713-0.885)	≤232.5 (81.6, 65.9)
3	0.726 (0.622-0.815)	≤48 (61.2, 85.4)	0.652 (0.544-0.749)	≤232.1 (59.2, 68.3)
4	0.703 (0.597-0.795)	≤53 (53.1, 85.4)	0.537 (0.428-0.642)	≤277.0 (40.8, 73.2)
5	0.629 (0.521-0.729)	≤66 (38.8, 92.7)	0.549 (0.440-0.654)	>450.8 (20.4, 95.1)
6	0.591 (0.482-0.693)	≤93 (30.6, 92.7)	0.603 (0.494-0.704)	>403.2 (40.8, 82.9)
7	0.639 (0.531-0.737)	≤147 (73.5, 51.2)	0.531 (0.423-0.637)	>271.4 (71.4, 41.5)
8	0.566 (0.458-0.670)	≤69 (38.8, 80.5)	0.591 (0.483-0.694)	>258.0 (42.9, 80.5)
9	0.585 (0.477-0.688)	≤58 (69.4, 46.3)	0.585 (0.476-0.688)	>210.5 (63.3, 68.3)

Statistically significant values are shown in bold. CI, confidence interval.

[a] The best cut-off value was selected using the Youden index value that maximized the value of 'sensitivity + specificity – 1'.

Supplementary Table S2. AUROC Curve Comparison of RNFLT and 3D-NRRT for Differential Diagnosis of NTG from Normal Eyes

	RNFLT		3D-NRRT	
	**AUROC (95% CI)**	**Best Cut-Off[a] (Sensitivity, Specificity)**	**AUROC (95% CI)**	**Best Cut-Off[a] (Sensitivity, Specificity)**
**Average**	0.871 (0.786-0.932)	≤88.3 (80.8, 82.9)	0.760 (0.661-0.843)	≤228.6 (55.8, 90.2)
**Quadrant**				
Superior	0.941 (0.872-0.979)	≤99 (90.4, 90.2)	0.883 (0.799-0.940)	≤211.8 (63.5, 100.0)
Nasal	0.647 (0.541-0.743)	≤54 (28.9, 100.0)	0.608 (0.501-0.708)	≤253.3 (50.0, 70.7)
Inferior	0.713 (0.610-0.802)	≤107 (61.5, 80.5)	0.702 (0.598-0.793)	≤258.2 (48.1, 87.8)
Temporal	0.691 (0.586-0.782)	≤68 (65.4, 70.7)	0.748 (0.647-0.832)	≤172.9 (59.6, 87.8)
**Clock hours**				
10	0.753 (0.652-0.836)	≤75 (61.5, 80.5)	0.763 (0.664-0.845)	≤145.4 (42.3, 100.0)
11	0.977 (0.923-0.997)	≤100 (90.4, 97.6)	0.908 (0.830-0.958)	≤215.5 (76.9. 95.1)
12	0.744 (0.643-0.829)	≤100 (69.2, 70.7)	0.870 (0.784-0.931)	≤248.8 (76.9, 85.4)
1	0.631 (0.525-0.729)	≤107 (86.5, 34.2)	0.737 (0.635-0.823)	≤242.2 (69.2, 78.1)
2	0.662 (0.556-0.756)	≤69 (53.9, 78.1)	0.649 (0.543-0.745)	≤300.4 (86.5, 43.9)
3	0.579 (0.472-0.680)	≤59 (71.2, 48.8)	0.605 (0.498-0.705)	≤273.4 (71.2, 48.8)
4	0.590 (0.483-0.691)	≤55 (38.5, 80.5)	0.563 (0.456-0.666)	≤276.8 (46.2, 73.2)
5	0.597 (0.490-0.697)	≤74 (44.2, 82.9)	0.59 (0.487-0.694)	≤296.1 (46.2, 78.1)
6	0.616 (0.509-0.715)	≤119 (71.2, 48.8)	0.695 (0.591-0.786)	≤313.1 (57.7, 75.6)
7	0.710 (0.607-0.800)	≤121 (44.2. 92.7)	0.773 (0.675-0.854)	≤228.4 (59.6, 87.8)
8	0.624 (0.518-0.722)	≤67 (42.3, 85.4)	0.730 (0.628-0.817)	≤163.4 (50.0, 95.1)
9	0.538 (0.431-0.642)	≤51 (40.4, 75.6)	0.698 (0.594-0.789)	≤195.9 (73.1, 63.4)

Statistically significant values are shown in bold. CI, confidence interval.

[a] The best cut-off value was selected using the Youden index value that maximized the value of ‘sensitivity + specificity – 1’.
